# NDR1 enhances USP9X-mediated AR deubiquitination and promotes enzalutamide resistance in castration-resistant prostate cancer

**DOI:** 10.7150/ijbs.114686

**Published:** 2025-09-03

**Authors:** Zeyuan Zheng, Jinxin Li, Yifan Du, Liyan Li, Qingqing Wu, Bin Liu, Haodong Wu, Zeyi Zhang, Zuodong Xuan, Yue Zhao, Huimin Sun, Chen Shao

**Affiliations:** 1 Department of Urology, Xiang'an Hospital of Xiamen University, School of Medicine, Xiamen University, Xiamen 361101, China.; 2 Department of Urology, Changhai Hospital, Naval Medical University (Second Military Medical University), Shanghai, China.; 3 Central Laboratory, Xiang'an Hospital of Xiamen University, School of Medicine, Xiamen University, Xiamen 361101, China.

**Keywords:** Castration-resistant prostate cancer, Enzalutamide resistance, AR, NDR1, USP9X

## Abstract

Castration-resistant prostate cancer (CRPC) enzalutamide resistance is a significant issue in the current treatment of prostate cancer (PCa). Previously, nuclear Dbf2-related 1 (NDR1) was found to influence metastasis in PCa patients; however, the role of NDR1 in enzalutamide resistance in CRPC remains unclear. In this study, we found that after CRPC cells developed resistance to enzalutamide, NDR1 expression levels were elevated and that NDR1 expression could reduce the sensitivity of CRPC cells to enzalutamide. Furthermore, in androgen receptor (AR) positive PCa cell lines, the use of enzalutamide induced an increase in NDR1 expression levels. Further mechanistic exploration revealed that NDR1 positively regulates AR protein expression levels by promoting the deubiquitination of AR by USP9X, thereby increasing AR stability, which leads to cellular resistance to enzalutamide. Finally, we confirmed that pharmacological suppression of NDR1 by 17AAG significantly inhibited the growth of enzalutamide-resistant CRPC tumors in both *in vitro* and *in vivo* models. In summary, this study revealed that NDR1 enhances the deubiquitination of AR mediated by USP9X, improving its stability and activity and thereby maintaining the continuous activation of the androgen signaling pathway in CRPC, leading to resistance to enzalutamide treatment. These findings suggest that cotargeting NDR1 and AR may represent a novel therapeutic strategy for AR-positive CRPC.

## Introduction

Prostate cancer (PCa) is the most common malignancy of the male urinary system and one of the top five causes of cancer-related death among men 1. Androgens play pivotal roles in the initiation and progression of PCa 2-4, making androgen deprivation therapy (ADT) the standard treatment for patients with PCa 5. Although ADT initially induces a response in more than 90% of patients, disease progression occurs at a median of 12-14 months despite testosterone suppression, at which point patients are diagnosed with castration-resistant PCa (CRPC) 6-8. CRPC is challenging to treat clinically because of inherent or acquired resistance, and despite a significant reduction in androgen levels, cancer continues to progress, making it a leading cause of cancer-related death 9. The androgen receptor (AR)-related signaling pathway is considered a critical mechanism underlying enzalutamide resistance in CRPC, including abnormal AR amplification and/or overexpression, AR mutations, and the generation of AR splice variants (AR-Vs) 10-12. Since AR's role in hormone dependence was first described in 1941, blocking AR signaling has been the cornerstone of PCa treatment 13.

Enzalutamide is a key second-generation AR inhibitor currently used to treat CRPC 14. It competitively binds to the ligand-binding domain of the AR, inhibiting androgen binding, AR nuclear translocation, and AR-mediated DNA binding, thereby effectively suppressing the progression of CRPC 15, 16. Compared with the placebo, enzalutamide significantly prolongs overall survival and progression-free survival in men with metastatic CRPC (mCRPC) who have previously received docetaxel 17, 18. However, approximately 42% of patients are unresponsive to enzalutamide in clinical practice, and even among those who initially respond, resistance may develop after a median of 11.2 months 19. The incidence and mortality rates of CRPC are increasing, and current treatment options are limited in their effectiveness in controlling the disease. Therefore, further investigation into the mechanisms of enzalutamide resistance, as well as the development of novel therapeutic strategies and exploration of innovative targeted therapies or drug combinations to prolong the survival of CRPC patients, has become an urgent task in medical research.

Nuclear Dbf2-related 1 (NDR1, also known as STK38) is a member of the NDR family and serves as an essential cell cycle regulatory protein, playing a critical role in biological processes such as cell division, proliferation, and apoptosis 20. Additionally, NDR1 has been implicated in the initiation and progression of tumors. It was previously thought to function primarily as a tumor suppressor within the context of the HIPPO pathway in the tumor microenvironment 21. However, recent research suggests that NDR1 may have multiple functions depending on the type of tumor or stage of tumor progression, and its role is not unidirectional. Under cellular stress conditions, NDR1 may exert protumorigenic effects 22. For example, NDR1 enhances the stability and nuclear localization of the ASCL47 protein, activating CD1 transcription, thereby promoting cancer stem cell characteristics and assisting small cell lung cancer in evading immune phagocytosis 23. Furthermore, NDR1 affects the stability of various proteins through protein‒protein interactions, with its kinase activity not being the sole critical factor 24. For example, NDR1, a novel cofactor of PPARγ, stabilizes PPARγ and promotes adipogenesis 25. In the context of tumor drug resistance, Wang et al. first reported that NDR1 competitively binds to the NICD with Fbw7, reducing NICD proteolytic degradation, thereby activating Notch signaling and promoting doxorubicin resistance in breast cancer 26. Previous studies have reported that NDR1 is associated with the development and progression of PCa 27, 28; however, the role of NDR1 in enzalutamide resistance in CRPC remains unclear.

In this study, we found that in the early stages of enzalutamide treatment for CRPC, AR is inhibited, which subsequently induces an increase in NDR1 protein expression. When NDR1 protein levels are elevated in CRPC cells, NDR1 promotes the expression of USP9X, which binds to and deubiquitinates AR, thereby increasing AR protein stability within the cell. These factors lead to the abnormal overexpression of AR in CRPC, resulting in resistance to enzalutamide. Further cell and animal experiments demonstrated that targeting NDR1 with inhibitors effectively reversed enzalutamide resistance in CRPC. In summary, this study reveals a potential novel mechanism of enzalutamide resistance in CRPC and provides a new therapeutic strategy to combat enzalutamide resistance in CRPC patients in clinical settings.

## Results

### NDR1 expression is elevated following the development of enzalutamide resistance in CRPC

**Figure 1:**
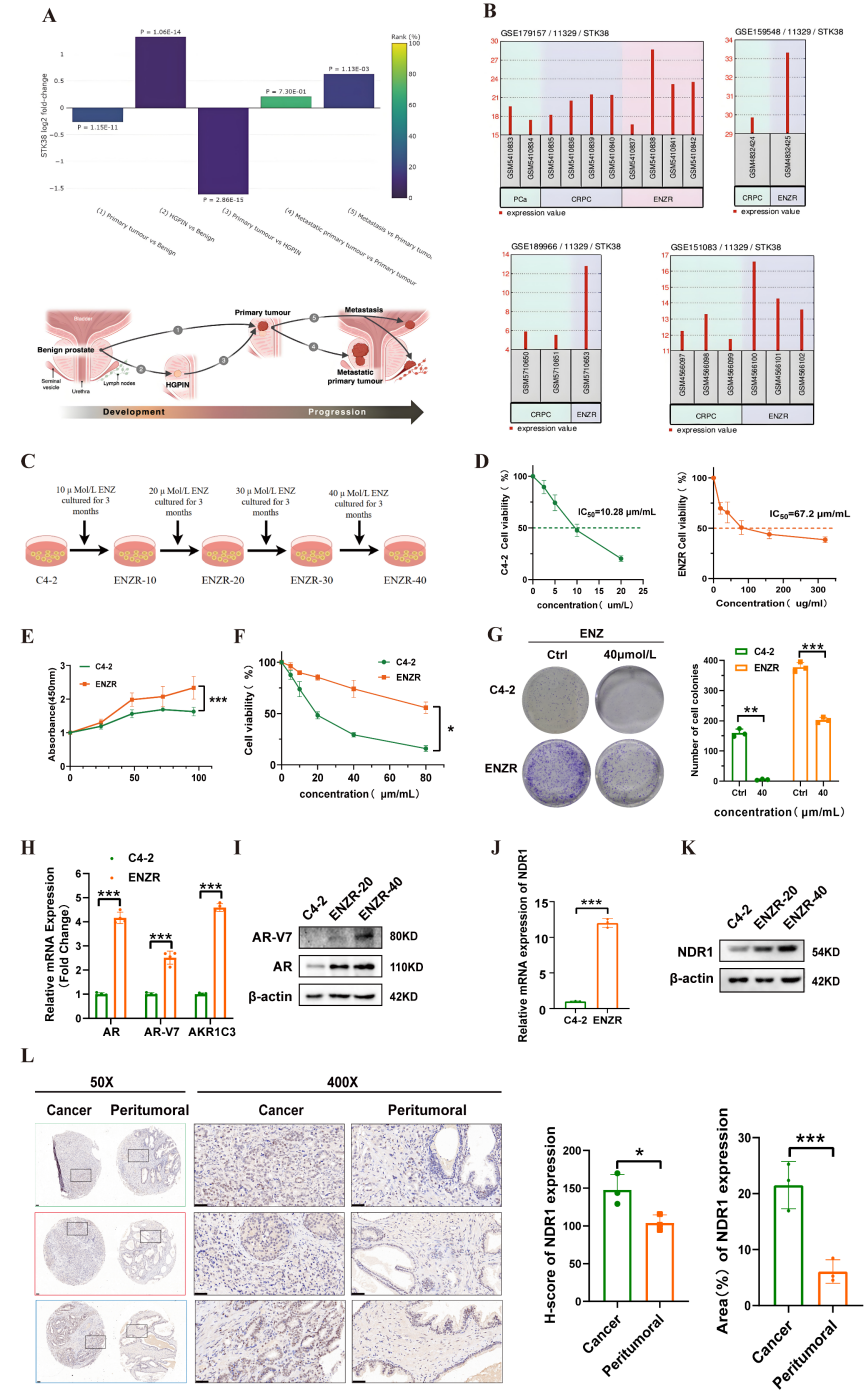
** NDR1 expression is elevated following the development of enzalutamide resistance in CRPC.** A: Bioinformatic prediction of STK38 (NDR1) expression across different stages of prostate cancer progression, including benign prostate, HGPIN, primary tumor, and metastatic lesions. B: Differential expression analysis of NDR1 in multiple GEO datasets (GSE179157, GSE119548, GSE189966, and GSE151083) demonstrating consistent upregulation in CRPC and ENZR samples. C: Schematic illustration of the stepwise generation of enzalutamide-resistant cell lines (ENZR-10, -20, -30, -40) from C4-2 cells by gradual exposure to increasing concentrations of enzalutamide over a 3-month period per stage. D: IC50 analysis of C4-2 and ENZR cells (n=3). E-F: Viability of C4-2 and ENZR cells (n=3). G: Colony formation assay showing ENZR cell resistance to enzalutamide (n=3). H: mRNA levels of AR, ARV7, and AKR1C3 in ENZR cells (n=3). I: Protein levels of AR and ARV7 in ENZR cells. J: NDR1 mRNA expression in ENZR cells (n=3). K: NDR1 protein levels in ENZR cells. L: Representative IHC images and statistical analysis of NDR1 expression in tumor tissues and matched adjacent noncancerous tissues from three AR-V7-positive prostate cancer patients. NDR1 shows predominant cytoplasmic localization in tumor cells. Quantitative analysis revealed significantly higher NDR1 expression in tumor regions compared to normal tissues (mean H-score: tumor = 185 ± 22 vs. normal = 45 ± 15; *p < 0.001, unpaired t-test). Scale bar: 50 μm. Error bars represent mean ± SD. (n=3) *p < 0.05; **p < 0.01; ***p < 0.001

We analyzed the Prostate Integrative Expression Database (PIXdb) and found significant differences in NDR1 expression across PCa stages, with levels changing during tumor progression **(Fig. 1A).** Further analysis of multiple GSE datasets (GSE179157, GSE159548, GSE189966, and GSE151083) revealed higher NDR1 expression in enzalutamide-resistant CRPC patients compared to PCa and CRPC patients** (Fig. 1B)**, suggesting its role in resistance development.

To investigate this, we established the enzalutamide-resistant (ENZR) cell line from C4-2 cells via a concentration gradient method** (Fig. 1C)**. ENZR cells exhibited an IC50 of 67.2 µmol/L, approximately 6.54 times that of C4-2 cells **(Fig. 1D)**. Viability assays showed enhanced proliferation and survival of ENZR cells under enzalutamide treatment **(Fig. 1E, F).** Colony formation assays further confirmed resistance, with ENZR cells maintaining growth under 40 µmol/L enzalutamide, while C4-2 cells were significantly inhibited **(Fig. 1G)**. RT‒qPCR and Western blot analyses showed elevated expression of resistance markers AR, AR-V7, and AKR1C3 at both mRNA and protein levels in ENZR cells **(Fig. 1H, I)**, confirming successful model establishment.

We next validated NDR1 expression, finding its mRNA levels increased in ENZR cells** (Fig. 1J)**, with progressive upregulation during resistance development **(Fig. 1K)**. Since AR-V7 is linked to resistance to second-generation AR inhibitors, we further examined prostate cancer tissues from three AR-V7-positive patients using IHC. Quantitative analysis revealed that NDR1 expression was significantly elevated in tumor tissues compared to matched adjacent noncancerous areas.

Notably, NDR1 displayed predominant cytoplasmic localization in tumor cells, a pattern consistent with its known function in regulating protein stability through cytoplasmic interactions, such as NDR1-mediated PD-L1 deubiquitination via USP10 in the cytoplasm 27 **(Fig. 1L)**. These findings suggest that NDR1 is not only upregulated in enzalutamide-resistant CRPC but may exert its pro-survival effects primarily through cytoplasmic modulation of key signaling components.

### NDR1 affects the sensitivity of CRPC cells to enzalutamide

**Figure 2:**
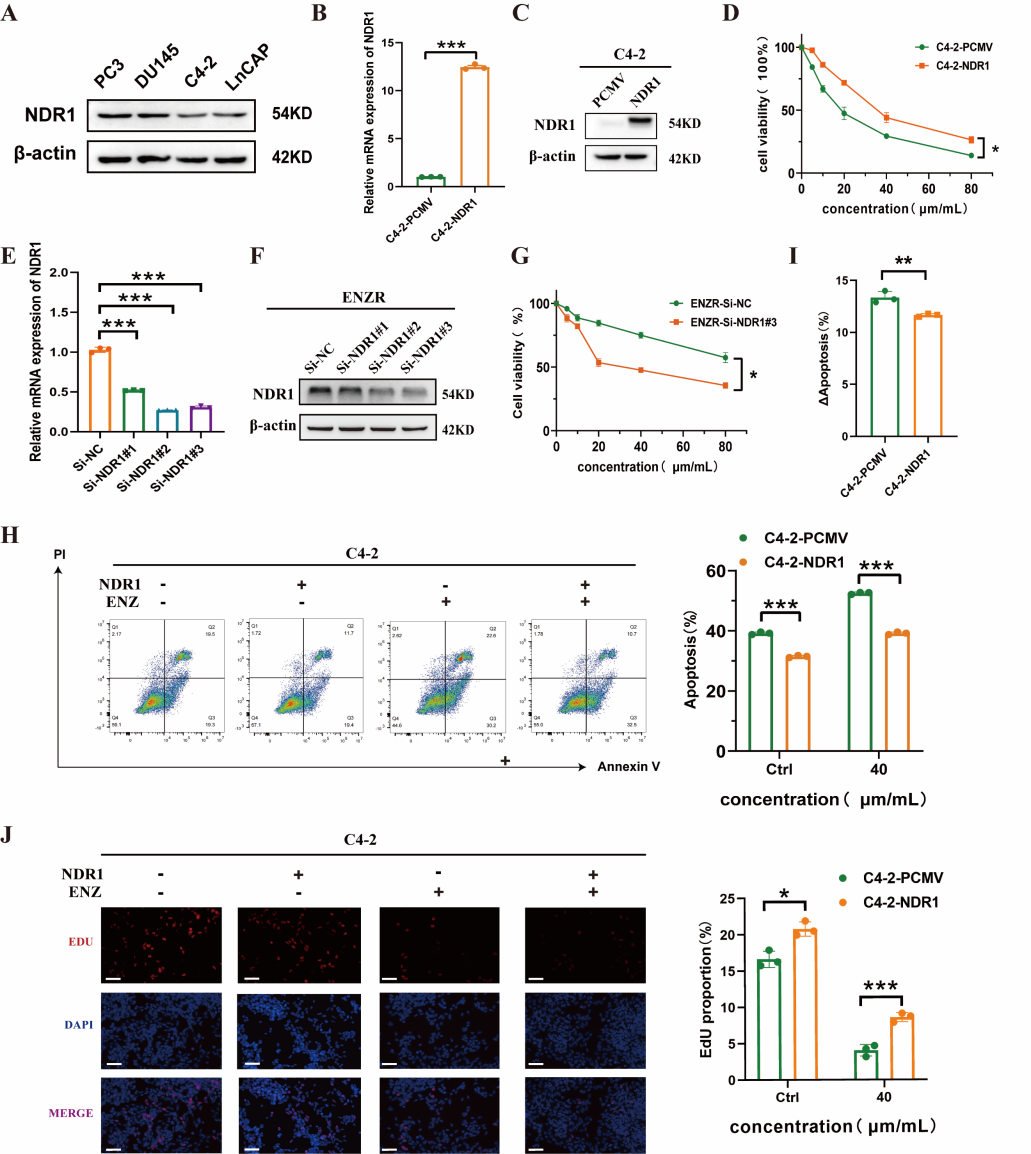
** NDR1 modulates enzalutamide sensitivity in CRPC cells.** A: Western blot analysis of NDR1 expression in PC3, DU145, C4-2, and LNCaP cells. B: qRT-PCR validation of NDR1 overexpression in C4-2 cells (n=3). C: Western blot confirmation of NDR1 protein overexpression in C4-2 cells. D: Cell viability assay showing increased survival of C4-2-NDR1 cells upon enzalutamide treatment (n=3). E: qRT-PCR analysis of NDR1 knockdown efficiency in ENZR cells using three different siRNAs (n=3). F: Western blot validation of NDR1 knockdown in ENZR cells. G: Cell viability assay showing that NDR1 knockdown sensitizes ENZR cells to enzalutamide (n=3). H: Flow cytometry analysis showing reduced apoptosis in C4-2-NDR1 cells after 10 µmol/L enzalutamide treatment compared to control (n=3). I: ΔApoptosis (%) calculated as the difference in apoptosis before and after enzalutamide treatment, showing attenuated apoptosis increase in C4-2-NDR1 cells (n=3). J: Representative EdU staining images and quantification showing that NDR1 overexpression enhances proliferation despite enzalutamide exposure (n=3). Scale bar: 50 μm. Error bars represent mean ± SD; *p < 0.05; **p < 0.01; ***p < 0.001.

To assess whether NDR1 influences the sensitivity of CRPC) cells to enzalutamide, we first evaluated its expression in prostate cancer cell lines. NDR1 protein was variably expressed among PC3, DU145, LNCaP, and C4-2 cells, with relatively low levels in C4-2 cells **(Fig. 2A)**. We established NDR1-overexpressing C4-2 cells and confirmed the overexpression at both mRNA and protein levels **(Fig. 2B**-**C)**. CCK8 assays showed that NDR1 overexpression significantly increased cell viability in response to enzalutamide treatment (Fig. 2D). Conversely, knockdown of NDR1 using siRNAs in ENZR cells (validated by RT-qPCR and Western blot in **Fig. 2E**-**F**) reduced cell viability under enzalutamide exposure **(Fig. 2G)**. To further evaluate the effect of NDR1 on drug sensitivity, we conducted Annexin V/PI staining. Overexpression of NDR1 suppressed enzalutamide-induced apoptosis in C4-2 cells **(Fig. 2H)**, and the increase in apoptosis upon drug treatment (ΔApoptosis) was significantly lower in NDR1-overexpressing cells than in controls **(Fig. 2I)**. Additionally, EdU incorporation assays demonstrated that NDR1 enhanced proliferative activity under enzalutamide treatment **(Fig. 2J)**.

Together, these data suggest that NDR1 overexpression promotes enzalutamide resistance in CRPC cells by inhibiting apoptosis and sustaining proliferation.

### Enzalutamide induces NDR1 expression in AR-positive CRPC cells

**Figure 3:**
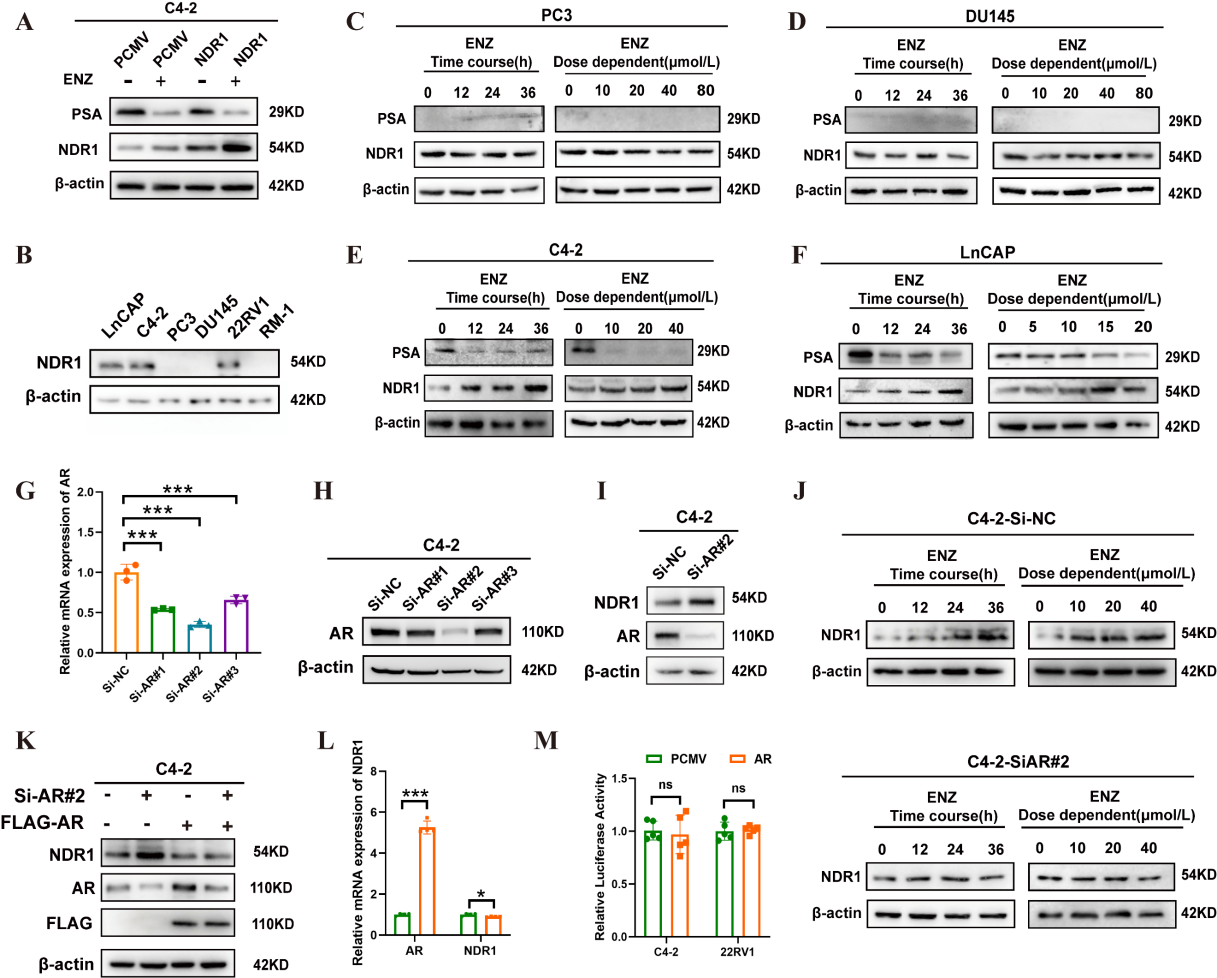
** Enzalutamide induces NDR1 expression in AR-positive CRPC cells.** A: Western blot analysis of NDR1 expression in C4-2 cells overexpressing NDR1 or vector control (PCMV) with or without enzalutamide (10 µmol/L) treatment. B: NDR1 protein expression in AR-positive (LNCaP, C4-2, 22RV1) and AR-negative (PC3, DU145, RM-1) CRPC cell lines. C-D: Time-course and dose-dependent effect of enzalutamide on NDR1 expression in AR-negative PC3 (C) and DU145 (D) cells. E-F: Time-course and dose-dependent effect of enzalutamide on NDR1 expression in AR-positive C4-2 (E) and LNCaP (F) cells. G-H: Validation of AR knockdown efficiency by qPCR (G) and western blot (H) in C4-2 cells transfected with siRNA (Si-AR#1-3). I: NDR1 expression following AR knockdown in C4-2 cells. J: Enzalutamide treatment in AR-knockdown C4-2 cells (Si-AR#2) did not significantly increase NDR1 expression. K: Rescue experiment showing re-expression of FLAG-AR reverses NDR1 upregulation in AR-knockdown C4-2 cells. L: qPCR analysis showing that AR overexpression reduces NDR1 mRNA levels (n=3). M: Dual-luciferase reporter assay indicates that AR overexpression does not affect NDR1 promoter activity in C4-2 or 22RV1 cells (n=5). Error bars represent mean ± SD; *p < 0.05; **p < 0.01; ***p < 0.001. ns, not significant.

To further investigate the mechanism underlying NDR1 upregulation, we treated NDR1-regulated C4-2 cells with enzalutamide and observed increased NDR1 expression **(Fig. 3A)**. Given that enzalutamide primarily targets AR, we examined AR status across CRPC cell lines **(Fig. 3B)**, classifying them as AR-positive (LNCaP, C4-2, 22RV1) or AR-negative (PC3, DU145, RM-1). Enzalutamide treatment elevated NDR1 levels only in AR-positive C4-2 and LNCaP cells in a time- and dose-dependent manner, but not in AR-negative PC3 or DU145 cells **(Fig. 3C**-**F)**, suggesting that AR status influences NDR1 response to enzalutamide.

We next tested whether AR negatively regulates NDR1. AR knockdown in C4-2 cells led to a significant increase in NDR1 expression** (Fig. 3G**-**I)**. Notably, enzalutamide treatment did not further elevate NDR1 in AR-silenced cells **(Fig. 3J)**, indicating that AR inhibition is necessary for enzalutamide-induced NDR1 upregulation. To confirm this, we performed AR rescue experiments. Re-expression of FLAG-AR in AR-knockdown C4-2 cells suppressed NDR1 protein and mRNA levels **(Fig. 3K**-**L)**, further supporting that AR negatively regulates NDR1.

However, dual-luciferase assays using a 2000 bp NDR1 promoter construct showed no significant change in luciferase activity upon AR overexpression** (Fig. 3M)**, suggesting that AR does not directly regulate NDR1 transcription. *In silico* analysis using the JASPAR database also failed to identify canonical AR binding motifs within the promoter region **(Fig. S1).** We therefore speculate that AR may act via an indirect mechanism, possibly by modulating an intermediate repressor of NDR1, which will be a key focus of our future investigations.

### NDR1 binds to and regulates AR expression

**Figure 4:**
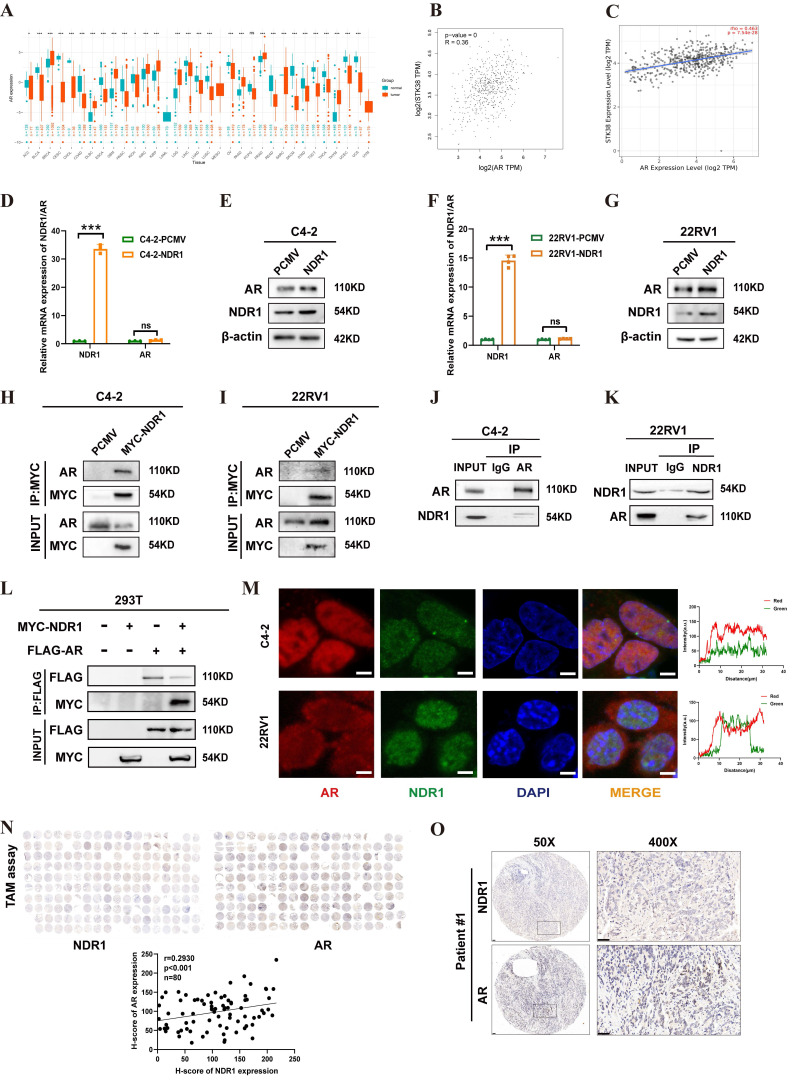
** NDR1 binds to and regulates AR expression.** A: Pan-cancer correlation analysis of AR mRNA expression across TCGA tumors. B-C: Positive correlation between NDR1 and AR mRNA levels in prostate cancer based on GEPIA2 (B) and TIMER2.0 (C). D-G: RT-qPCR and WB analysis of NDR1 and AR in C4-2 (D-E) and 22RV1 (F-G) cells after NDR1 overexpression (n=3). H-I: Co-IP and WB showing AR-NDR1 interaction in C4-2 (H) and 22RV1 (I) cells after NDR1 overexpression. J-K: Endogenous Co-IP detecting AR-NDR1 binding in C4-2 (J) and 22RV1 (K) cells. L: Co-IP and WB confirming AR-NDR1 interaction in 293T cells overexpressing NDR1 and AR. M: Immunofluorescence staining of AR in red, NDR1 in green, and DAPI in blue in C4-2 and 22Rv1 cells. NDR1 shows punctate nuclear distribution with partial co-localization with AR. Line-scan analysis (right) confirms signal overlap in several nuclear regions. (scale bar: 5μm). N: Tissue microarray IHC analysis of NDR1 and AR expression in prostate cancer (n=80), showing a positive correlation. O: Representative IHC images of NDR1 and AR staining in one patient. Scale bars: 50 μm (left), 400× magnification (right). Error bars represent mean ± SD; *p < 0.05; **p < 0.01; ***p < 0.001; ns, not significant.

AR plays a key role in PCa development and is highly expressed in PCa tissues** (Fig. 4A)**. To investigate its correlation with NDR1, we analyzed GEPIA and TIMER2.0 databases, revealing a positive association **(Fig. 4B-C)**. Overexpressing NDR1 in C4-2 and 22RV1 cells significantly increased AR protein levels **(Fig. 4D-G)** but had no effect on AR mRNA expression** (Fig. 4D-G)**, suggesting post-transcriptional regulation.

Co-immunoprecipitation (Co-IP) in C4-2 and 22RV1 cells confirmed NDR1-AR binding **(Fig. 4H-K)**. Exogenous Co-IP in HEK293T cells transfected with MYC-NDR1 and/or FLAG-AR further demonstrated a direct interaction at the protein level** (Fig. 4L)**. Immunofluorescence staining showed that both NDR1 and AR are mainly localized in the nucleus of C4-2 and 22Rv1 cells, with partially overlapping signals. NDR1 displayed a punctate nuclear pattern, particularly in 22Rv1 cells. Line-scan analysis confirmed partial nuclear co-localization of NDR1 and AR **(Fig. 4M)**. These findings indicate that NDR1 regulates AR at the protein level and directly interacts with AR.

To validate these results, we analyzed human tissue microarrays from 80 PCa patients. Immunohistochemical staining showed NDR1 expression in cancer tissues correlated with AR levels **(Fig. 4N-O)**, further confirming a positive association between NDR1 and AR in PCa.

### NDR1 promotes AR protein stability

**Figure 5:**
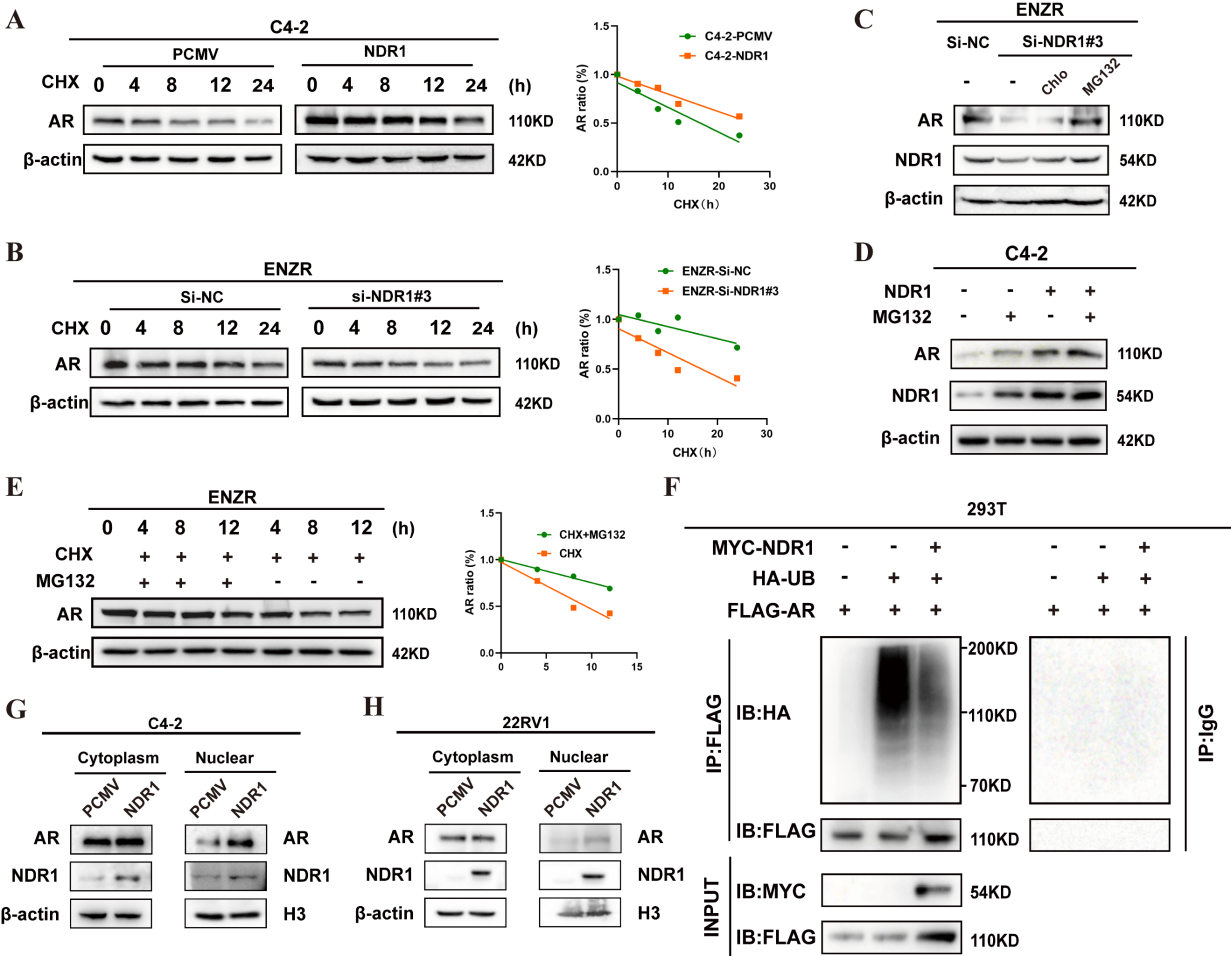
** NDR1 promotes AR stability by inhibiting its ubiquitination.** A: Analysis of AR protein stability in C4-2-PCMV and C4-2-NDR1 cells treated with CHX (100 µg/ml) at specified time intervals. B: Analysis of AR protein stability in ENZR-Si-NC and ENZR-Si-NDR1#3 cells treated with CHX (100 µg/ml) at specified time intervals. C: AR and NDR1 protein levels in ENZR-Si-NDR1#3 cells treated with DMSO, CQ, or MG132. D: AR and NDR1 protein levels in C4-2 cells overexpressing NDR1 and treated with MG132(40 µg/ml). E: AR expression analysis in ENZR cells treated with CHX with or without MG132, with quantification of AR degradation kinetics. F: Exogenous IP experiment in 293T cells showing that NDR1 overexpression reduces AR ubiquitination. IgG-IP was included as a negative control; molecular weights and AR band position (~110 kDa) are indicated. G-H: Expression levels of nuclear and cytoplasmic AR and NDR1 proteins in C4-2 (G) and 22Rv1 (H) cells were assessed by subcellular fractionation. H3 and β-actin were used as nuclear and cytoplasmic markers, respectively.

Targeting AR for degradation is a central theoretical foundation for the development of AR-inhibiting drugs 29. Given that NDR1 regulates AR at the protein level, we investigated its role in AR protein stability. Cycloheximide (CHX) treatment of NDR1-overexpressing C4-2 cells significantly enhanced AR stability, while NDR1 knockdown in ENZR cells reduced AR stability **(**quantified in right panels, **Fig. 5A-B)**.

Inhibition assays with MG132 (ubiquitin‒proteasome inhibitor) and chloroquine (autophagy‒lysosome inhibitor) showed that MG132 partially rescued AR levels in NDR1-knockdown ENZR cells, whereas chloroquine had no effect** (Fig. 5C)**. Similarly, both NDR1 overexpression and MG132 treatment increased AR protein levels in C4-2 cells **(Fig. 5D)**. Notably, MG132 also slightly elevated NDR1 levels in vector control cells, suggesting that NDR1 itself may be partially regulated via proteasomal degradation.

Time-course MG132 treatment (4 h, 8 h, 12 h) confirmed that NDR1 regulates AR through the ubiquitin‒proteasome pathway **(**quantified on the right, **Fig. 5E)**. Ubiquitination assays in 293T cells further revealed that NDR1 overexpression reduced AR ubiquitination, stabilizing AR **(Fig. 5F)**.

Additionally, nuclear-cytoplasmic fractionation assays demonstrated increased nuclear AR accumulation upon NDR1 overexpression** (Fig. 5G-H)**, suggesting NDR1 facilitates AR nuclear translocation. This may result from enhanced AR stability, prolonging cytoplasmic retention and increasing nuclear translocation under androgen stimulation. Future studies will further elucidate the regulatory mechanisms by which NDR1 stabilizes AR.

### NDR1 Regulates AR Stability via USP9X in CRPC enzalutamide resistance

**Figure 6:**
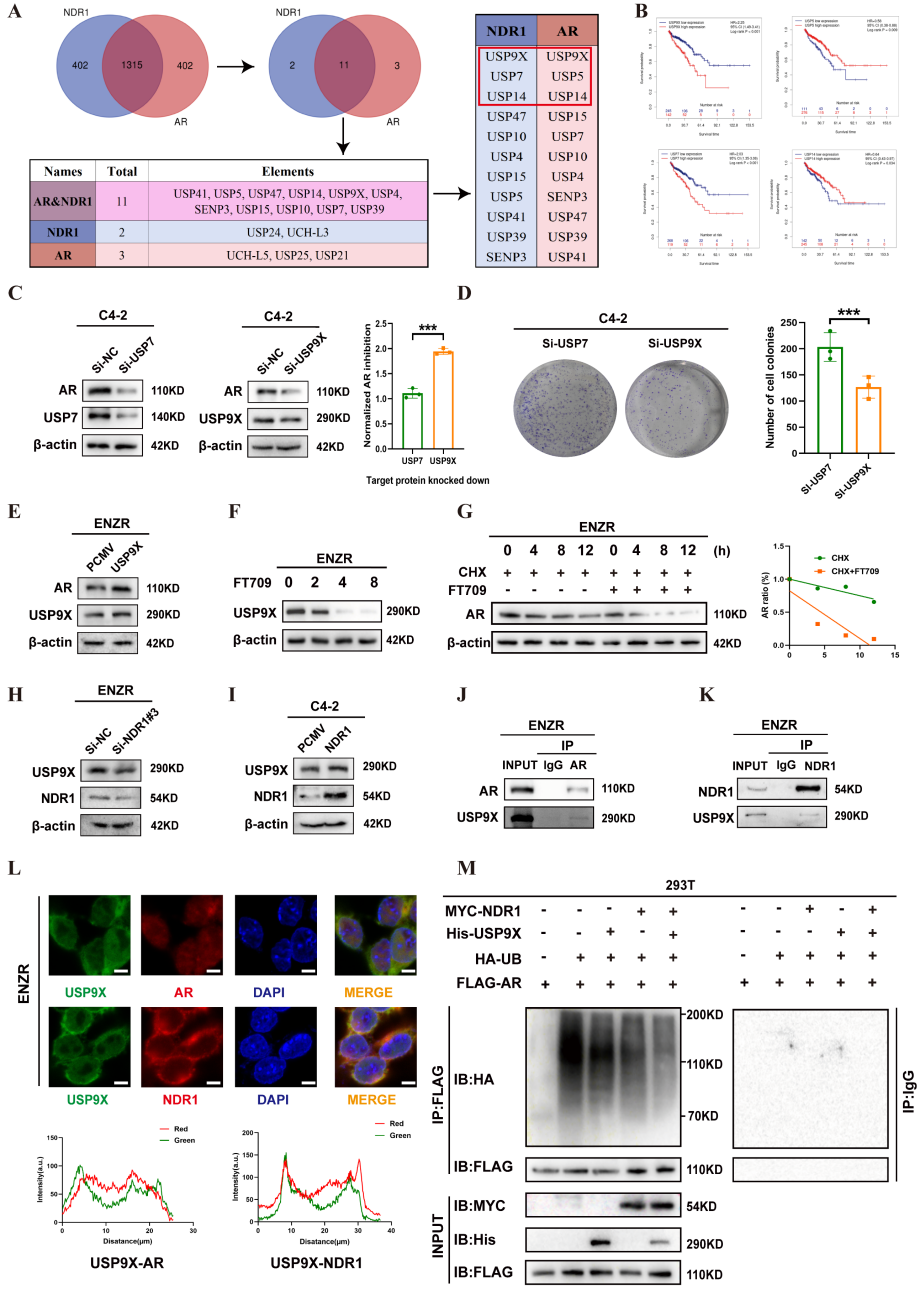
** NDR1 upregulates USP9X to promote AR stabilization via deubiquitination.** A: Co-IP-MS analysis identified 11 deubiquitinases shared by NDR1 and AR, with USP9X, USP7, and USP14 among the top hits. B: TIMER 2.0 analysis showed that high USP9X expression was associated with poor prognosis in PCa. C: AR expression after knockdown of USP7 or USP9X in C4-2 cells. (n=3) D: Colony formation assay after knockdown of USP7 or USP9X in C4-2 cells. (n=3) E: AR and USP9X expression after USP9X overexpression in ENZR cells. F: ENZR cells were treated with increasing concentrations of FT709 (0, 2, 4, and 8 μmol/L) for 36 hours. Western blot analysis revealed that USP9X protein expression decreased progressively with higher FT709 doses, confirming effective inhibition of USP9X by FT709. G: CHX chase assay showing AR degradation rate with or without FT709 treatment. H: USP9X expression after NDR1 knockdown in ENZR cells. I: USP9X expression after NDR1 overexpression in C4-2 cells. J-K: Endogenous Co-IP showing interactions of USP9X with AR (J) or NDR1 (K) in ENZR cells. L: Immunofluorescence staining showing colocalization of USP9X with AR or NDR1 in ENZR cells (scale bar: 5 μm). M: Ubiquitination assay of AR in 293T cells. Cells were transfected with FLAG-AR, HA-ubiquitin, and MYC-NDR1 and/or His-USP9X. AR was immunoprecipitated using anti-FLAG or control IgG antibody and blotted with anti-HA. Error bars represent mean ± SD; *p < 0.05; **p < 0.01; ***p < 0.001; ns, not significant.

To explore how NDR1 stabilizes AR, we first performed Co-IP-MS in NDR1- and AR-overexpressing C4-2 cells, identifying 16 shared deubiquitinating enzymes (DUBs), among which USP5, USP7, USP9X, and USP14 ranked highest based on peptide scores **(Fig. 6A)**. By integrating TIMER 2.0 survival data and expression correlation analyses, we excluded USP5 and USP14 due to their association with favorable prognosis and weaker relevance to AR and NDR1** (Fig. 6B, Fig. S2A-D).**

This narrowed the candidates to USP7 and USP9X. Functional assays showed that USP9X knockdown caused greater AR reduction than USP7 after normalizing for knockdown efficiency **(Fig. 6C)**, and more significantly suppressed colony formation **(Fig. 6D)**. These findings supported USP9X as the key DUB mediating NDR1-dependent AR stabilization and justified its selection for further study.

Next, we examined the NDR1-USP9X regulatory relationship. In ENZR cells, USP9X overexpression led to a marked increase in AR protein levels** (Fig. 6E)**, while the USP9X-selective inhibitor FT709 induced a dose-dependent decrease in USP9X and AR expression** (Fig. 6F)**. Cycloheximide chase assays demonstrated that FT709 accelerated AR degradation, confirming the role of USP9X in maintaining AR stability** (Fig. 6G)**. Mechanistically, NDR1 knockdown reduced USP9X protein expression, while NDR1 overexpression enhanced USP9X levels **(Fig. 6H-I)**. Co-IP assays demonstrated that USP9X interacts directly with both AR and NDR1 **(Fig. 6J-K)**. Immunofluorescence further confirmed that USP9X co-localizes with AR and NDR1 in the cytoplasm of ENZR cells** (Fig. 6L)**, supporting their close spatial proximity in the deubiquitination machinery.

To determine whether NDR1 stabilizes AR through USP9X-mediated deubiquitination, we conducted a series of ubiquitination assays in 293T cells. As shown in the **Fig. S3A**, overexpression of USP9X significantly reduced the ubiquitination level of AR, confirming its direct role in AR deubiquitination. Subsequently we co-transfected MYC-NDR1 and His-USP9X with FLAG-tagged AR and HA-ubiquitin in 293T cells. As shown in **Figure 6M**, while USP9X or NDR1 individually decreased AR ubiquitination to varying degrees, co-expression of both proteins led to the most pronounced reduction in ubiquitination. Collectively, these results indicate that NDR1 acts not as a deubiquitinase, but as a facilitator of USP9X-mediated AR deubiquitination, by stabilizing USP9X and enhancing its binding to AR.

### NDR1 inhibitors play a role in reversing enzalutamide resistance in CRPC

**Figure 7:**
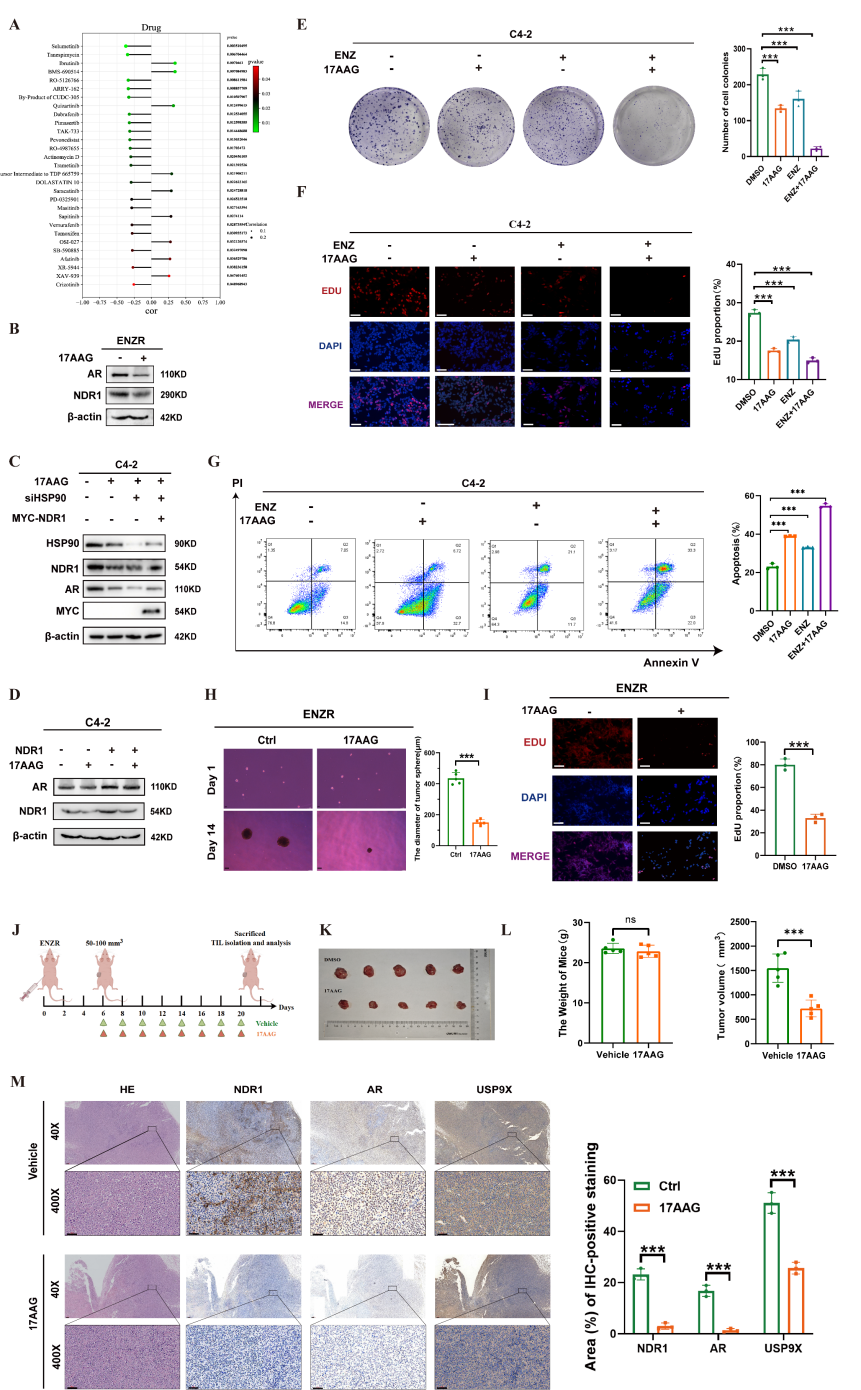
** The role of the NDR1 inhibitor in reversing enzalutamide resistance in CRPC.** A: Drug sensitivity profiling from the CellMiner database identifying candidate compounds negatively correlated with NDR1 expression. B: Western blot showing that 17AAG reduces AR and NDR1 expression in ENZR cells. C: Rescue experiment showing that NDR1 overexpression partially restores AR protein levels in HSP90-inhibited C4-2 cells. D: Western blot confirming that 17AAG reduces both endogenous and exogenous NDR1 protein expression. E-F: Colony formation (E) and EdU assays (F) showing reduced proliferation in C4-2 cells treated with enzalutamide and/or 17AAG (scale bar: 50 μm, n = 3). G: Flow cytometry revealing increased apoptosis after enzalutamide and/or 17AAG treatment in C4-2 cells (n = 3). H-I: Tumorsphere (H) and EdU (I) assays confirming that 17AAG alone reduces proliferation and tumorsphere formation in ENZR cells (scale bars: 150 μm in H, 50 μm in I, n = 3). J: Schematic illustration of the *in vivo* experimental workflow. K-L: Tumor images (K), body weights, and tumor volumes (L) showing that 17AAG inhibits tumor growth without affecting body weight in xenograft models (n = 5). M: H&E and IHC staining of xenograft tumors demonstrating decreased NDR1, AR, and USP9X expression upon 17AAG treatment (scale bar: 50 μm, n = 3). Error bars represent mean ± SD; *p < 0.05; **p < 0.01; ***p < 0.001; ns, not significant.

Building on previous findings, we investigated the link between NDR1 and drug sensitivity in CRPC. Using the CellMiner database, we identified 20 small-molecule compounds whose activity was significantly correlated with NDR1 expression **(Fig. 7A)**. Among these, tanespimycin (17-AAG), a known HSP90 inhibitor, was selected for further study based on prior research indicating its ability to reduce NDR1 expression. Treatment of ENZR cells with 17AAG markedly decreased both NDR1 and AR expression **(Fig. 7B)**. Consistently, overexpression of NDR1 partially rescued AR protein levels under HSP90 knockdown conditions, suggesting that the effect of 17AAG on AR is, at least in part, mediated through NDR1 **(Fig. 7C)**. Moreover, overexpression of NDR1 counteracted the suppressive effect of 17AAG on AR expression, confirming the involvement of NDR1 in this regulatory axis **(Fig. 7D)**.

Functional assays revealed that 17AAG enhanced the inhibitory effects of enzalutamide on C4-2 cell proliferation and viability, as shown by colony formation and EdU incorporation assays **(Fig. 7E-F)**, and significantly promoted apoptosis **(Fig. 7G)**. Tumor sphere formation assays further demonstrated that 17AAG reduced the size of ENZR-derived spheroids **(Fig. 7H)**, and EdU staining confirmed its inhibitory effect on proliferation **(Fig. 7I)**.

*In vivo*, xenograft experiments showed that 17AAG treatment led to a significant reduction in tumor volume without affecting body weight **(Fig. 7J-L)**, indicating favorable therapeutic efficacy. H&E staining of major organs including liver, heart, lung, kidney, and spleen revealed no significant histological abnormalities, suggesting low systemic toxicity** (Fig. S4A)**. Immunohistochemical analysis further confirmed that 17AAG downregulated NDR1, AR, and USP9X protein levels in tumor tissues, consistent with the *in vitro* results **(Fig. 7M)**.

Collectively, these data demonstrate that 17AAG exerts anti-tumor effects in CRPC, at least in part through NDR1 inhibition, and support its potential utility in overcoming enzalutamide resistance.

In summary, we propose a mechanistic model illustrating how NDR1 influences AR protein stability, thereby leading to enzalutamide resistance in CRPC, as shown in **Fig. S4B**. During the early stages of enzalutamide treatment in CRPC patients, enzalutamide inhibits AR activity, which induces the upregulation of NDR1 protein expression. As NDR1 protein levels increase in CRPC cells, NDR1 promotes the expression of USP9X, which binds to AR and deubiquitinates it, enhancing AR stability within the cell. Consequently, AR becomes abnormally overexpressed in CRPC cells, ultimately leading to resistance to enzalutamide.

## Discussion

As a second-generation androgen receptor signaling inhibitor (ARSI), enzalutamide has offered hope to patients with CRPC, as it significantly prolongs overall survival. However, the emergence of resistance has become a major clinical challenge in the treatment of CRPC, making the study of enzalutamide resistance mechanisms a key focus of current research. Mechanisms such as the AR signaling pathway 30, glucocorticoid receptor-related pathways 31, neuroendocrine transdifferentiation 32, and the activation of pathways such as the WNT/β-catenin, PI3K/AKT, and ERK1/2 33-35 pathways have all been implicated in mediating ENZR. Among these, the role of AR in CRPC enzalutamide resistance is unequivocal. A major research focus has been the relationship between protein homeostasis and AR, with ongoing exploration of strategies targeting AR protein stability or transcription levels to overcome CRPC enzalutamide resistance 36. For example, traditional drugs such as niclosamide have been repurposed, and several newly developed small-molecule compounds, such as MTX-23 37, BWA-522 38, UT-34 39, and AR-targeted PROTACs 40, have shown promising therapeutic efficacy. Despite the encouraging data, these drugs remain in the preclinical stage, with no treatments yet approved for clinical use to address enzalutamide resistance in CRPC.

NDR1 has been shown to influence various cellular biological processes 41. In tumor research, it has been widely recognized that NDR1 functions as a key component of the HIPPO signaling pathway. 42. Interestingly, increasing evidence suggests that NDR1 may be regulated differently across various tumor types. For instance, NDR1 mRNA expression levels are increased in progressive ductal breast carcinoma 43, lung adenocarcinoma 44, and ovarian cancer 45, whereas the NDR1 protein is highly expressed in some human melanoma cell lines 46. However, NDR1 expression is downregulated in gastric cancer 47, cutaneous squamous cell carcinoma 48, and acute lymphoma 49. Recent findings suggest that NDR1 also plays a role in the development of resistance to cancer therapies. It has been reported that NDR1 promotes resistance to epirubicin in breast cancer. 26, which, to our knowledge, is the first report on the involvement of NDR1 in cancer drug resistance. Our study reveals that NDR1 plays a role in enzalutamide resistance in CRPC and provides an in-depth exploration of the underlying mechanisms. This adds valuable insight to the existing body of research in this area.

In recent years, we have explored various aspects of NDR1's role in the progression of prostate cancer. Liu et al. demonstrated that NDR1 expression exhibits significant prognostic value in patients with different types of cancer and is closely related to cancer immunity 50, highlighting its multifaceted nature across tumor types and disease stages. Strikingly, emerging evidence reveals that NDR1's function is highly context-dependent. In AR-independent metastatic PCa, Xuan et al. showed that NDR1 phosphorylation suppresses β-catenin via FBXO11, inhibiting WNT-driven progression 28, 51, whereas Fu et al. revealed its pro-tumorigenic role in immune evasion through PD-L1 stabilization 27. A recent study reported that NDR1 agonism suppresses tumor growth in early-stage or AR-negative PCa models 52, our findings in advanced CRPC demonstrate a diametrically opposed role: enzalutamide-induced NDR1 upregulation stabilizes AR via USP9X-mediated deubiquitination, directly driving therapy resistance. This functional duality is further exemplified by its stage-specific interactions with signaling pathways. Thus, NDR1 operates as a molecular switch, exerting anti- or pro-tumor effects contingent on AR status, microenvironmental cues, and therapeutic pressure. These observations underscore the need for precision targeting strategies—agonism in AR-negative/early-stage disease versus inhibition in AR-positive CRPC—to align with its divergent roles. Within this framework, our study specifically addresses how NDR1 contributes to enzalutamide resistance in CRPC, a critical unmet challenge rooted in persistent AR signaling activation.

In this study, we established the enzalutamide-resistant cell line ENZR from C4-2 cells using a drug concentration escalation method. The results showed that NDR1 expression was elevated in ENZR cells, and enzalutamide treatment led to increased NDR1 expression in AR-positive CRPC cells, potentially due to AR inhibition. This suggests that during the development of enzalutamide resistance, upregulation of NDR1 expression may contribute to resistance, possibly through its interaction with AR. Mechanistic investigations revealed that NDR1 stabilizes AR protein levels, promoting resistance to enzalutamide in C4-2 cells. We found that NDR1 regulates AR protein stability through deubiquitination, and mass spectrometry identified USP9X as a key deubiquitinating enzyme involved in this process. NDR1 promotes USP9X-mediated deubiquitination of AR, leading to increased AR stability and resistance to enzalutamide.

Recent studies have shown that the multifunctional ubiquitin ligase SOCS2 induces NDR1 degradation, which may serve as a regulatory switch in the TNFα-NF-κB pathway, with NDR1 also being capable of enhancing NF-κB activity 53. Whether in the HIPPO pathway or in interactions with other molecules or pathways, limiting NDR1 expression to inhibit its activation or directly inhibiting its activation represents a key approach to neutralizing NDR1 function. Therefore, we screened for compounds that modulate NDR1 and, supported by previous research, identified 17AAG as an agent that indirectly suppresses NDR1 expression. 54. Phenotypic assays and mouse experiments further confirmed that 17AAG significantly suppressed the growth of enzalutamide-resistant CRPC tumors and reversed enzalutamide resistance in CRPC enzalutamide-resistant cells. These findings indicate that inhibiting NDR1 could have therapeutic potential for patients with enzalutamide-resistant CRPC.

In conclusion, our study reveals a novel role of NDR1 in promoting enzalutamide resistance in CRPC by enhancing USP9X-mediated deubiquitination and stabilization of full-length AR (AR-FL). This function appears closely tied to AR expression status, suggesting NDR1 as a potential therapeutic and prognostic target in AR-positive CRPC. While our findings primarily focus on AR-FL, preliminary data indicate that NDR1 does not directly interact with the AR splice variant AR-V7, though whether NDR1 indirectly modulates AR-V7 activity remains an open question **(Fig S3B)**.

Beyond its role in regulating AR stability, NDR1 may participate in broader oncogenic networks. Given that NDR1 is a serine/threonine kinase capable of interacting with AR, it is plausible that it may also influence AR phosphorylation or nuclear translocation—key events in CRPC progression—which warrants further investigation.

Although 17AAG demonstrated promising antitumor activity by downregulating NDR1, it is not a selective NDR1 inhibitor. Future efforts should aim to develop specific small-molecule inhibitors or PROTAC degraders targeting NDR1 to improve therapeutic efficacy against enzalutamide-resistant CRPC and potentially other NDR1-driven malignancies. While our current clinical observations support the upregulation of NDR1 in AR-V7-positive CRPC tissues, further validation in larger clinical cohorts will be essential to confirm its prognostic and therapeutic relevance.

## Conclusion

This study reveals how enzalutamide suppresses AR and induces NDR1 upregulation in the early phase of treatment in CRPC. Elevated NDR1 promotes USP9X expression, which stabilizes AR through deubiquitination, leading to AR overexpression and enzalutamide resistance. This mechanism was explored through cell lines, animal models, and clinical samples. Additionally, targeting NDR1 with inhibitors showed potential for treating enzalutamide-resistant CRPC. In summary, this research highlights that targeting NDR1 or USP9X could provide a promising strategy to overcome enzalutamide resistance.

## Materials and Methods

### Cell lines, cell culture, and construction of drug-resistant cells

Human PCa cell lines (LNCaP, C4-2, 22RV1, PC3, and DU145) and human kidney epithelial cells (293T) were purchased from the American Type Culture Collection (ATCC). C4-2, LNCaP, 22RV1, PC3, and DU145 cells were cultured in RPMI-1640 medium (GIBCO, USA) supplemented with 10% fetal bovine serum (FBS). 293T cells were cultured in DMEM (GIBCO, USA) supplemented with 10% FBS. All of the above cell lines were cultured with 100 U/mL penicillin and 100 µg/mL streptomycin in a humidified atmosphere of 5% CO2 at 37 ºC. Enzalutamide resistance in C4-2 cells was induced via a stepwise dose escalation method, starting at low concentrations and gradually increasing. Parental C4-2 cells in good growth conditions were initially cultured with 10 µmol/L enzalutamide (MCE, #HY-70002) for 3 months to establish an enzalutamide-resistant cell line at 10 µmol/L (referred to as ENZR-10). Some cells were cryopreserved, and the IC50 was assessed via the CCK8 assay. The culture was continued with 20 µmol/L enzalutamide for another 3 months, and the process was repeated until the resistant cell line could stably proliferate in 40 µmol/L enzalutamide medium. The final resistant cell line was named ENZR.

### RNA extraction and RT‒PCR

Total RNA was extracted from cells using the SPARKeasy Tissue/Cell RNA Rapid Extraction Kit (Shandong Sparkjade Biotechnology Co., Ltd., Cat# AC0201) or via TRIzol RNA isolation reagents (Thermo Fisher, #15596018) according to the manufacturer's instructions. General gene reverse transcription was carried out via the PrimeScript™ RT Master Mix (Clontech Laboratories, USA). Real-time PCR was performed via Hieff® qPCR SYBR Green Master Mix (No Rox) (Yeasen, #11201ES08) on a CFX96 deep-well real-time fluorescence PCR detection system (Bio-Rad, USA). The sequences of all the specified primers are listed in Supplementary Table 1.

### Protein extraction and western blotting

The cells were lysed in RIPA buffer (KeyGEN BioTECH), and the protein concentrations were quantified via the Bradford protein assay (KeyGEN BioTECH). The lysates were then subjected to SDS‒PAGE and transferred onto PVDF membranes (Millipore). The membranes were incubated with primary antibodies overnight at 4 ºC. The following primary antibodies were used in the experiments: anti-β-actin (HUABIO, #HA722023; Cell Signaling Technology, #4967), anti-NDR1 (Santa Cruz Biotechnology, #sc-365555; ABclonal, #A8191), anti-AR (Cell Signaling Technology, #5153; Proteintech, #81844-1-RR), anti-AR-V7 (Cell Signaling Technology, #19672), anti-PSA (Cell Signaling Technology, #5365), anti-USP7 (Proteintech, #66514-1-Ig), anti-USP9X (Proteintech, #55054-1-AP), anti-HA (Abcam, #ab137321), anti-HIS (Abcam, #ab18184), anti-Myc (Abcam, #ab9106), and anti-Flag (Sino Biological, #109143-MM13). Chemiluminescent signals were detected via the SuperSignal™ ECL Western Blotting Detection Reagent (Merck). Nuclear and cytoplasmic proteins were extracted using a Nucleoplasmic Protein Extraction Kit (Solarbio, #EX1420).

### Cell transfection

The plasmids were extracted using the SPARKeasy Endotoxin-Free Plasmid Midiprep Kit (Shandong Sparkjade Biotechnology Co., Ltd., #AD0107) according to the manufacturer's instructions. Plasmid DNA and SiRNA transfections, either individually or in combination, were performed via Lipofectamine 3000 (Thermo Fisher, USA). The plasmids and small interfering RNAs (siRNAs) used in this study were as follows: pCMV3-STK38-Myc (HG12319-NM, Sino Biological), pCMV3-AR-FLAG (HG29832-CF, Sino Biological), His-USP9X (HG19122-UT, Sino Biological), and HA-Ub (Addgene). Sequences of all the SiRNA are listed in Supplementary Table 2.

### Immunoprecipitation

Antibody dilutions were added to the protein lysate, which was subsequently incubated overnight at 4 ºC. Protein A/G magnetic beads were washed and equilibrated with bead washing buffer, washed three times, and stored at 4 ºC for later use. After equilibration, 40 µL of beads were added to the IP lysate, which had been incubated overnight, and the mixture was further incubated for 2-4 hours. The beads were then collected and washed three times with bead washing buffer. After the wash buffer was removed, 40 µL of loading buffer (2×) was added, and the sample was heated at 100 ºC for 10 minutes before analysis by Western blotting.

### Protein stability assay

Cycloheximide (CHX, MCE, #HY-12320) was added to the cells at a concentration of 100 µg/mL to inhibit protein synthesis, with the first group labeled 24 h. The cells were then cultured, and every 4 h, an equal amount of CHX was added to a separate group of cells, which were labeled as 12 h, 8 h, and 4 h, until the last group was labeled as 0 h. After the proteins were collected, Western blot analysis was performed to assess changes in protein expression levels over time.

### LC‒MS/MS

After enzymatic digestion of the protein bands, the peptides were extracted and detected via a timsTOF Pro mass spectrometer (Bruker) for tandem mass spectrometry (LC‒MS/MS) analysis. Database searches were conducted via Mascot v2.3.02 software. The LC‒MS/MS analysis was carried out at the Analytical Testing Center, School of Life Sciences, Xiamen University. (We thank Yaying Wu, Zheni Xu and Dr. C.C. Xie for mass spectrometry experiments and data analysis).

### Animal model

Six-week-old male athymic BALB/c nude mice were purchased from the Experimental Animal Center of Xiamen University (Xiamen, China) and maintained under specific pathogen-free (SPF) conditions. All animal procedures were reviewed and approved by the Xiamen University Laboratory Animal Center (Ethics No. XMULAC20200039). To establish xenograft tumors, ENZR cells (1 × 10⁶ cells/mouse) were subcutaneously injected into the dorsal flanks of the mice. Once tumors reached a volume of 50-100 mm^3^, mice were randomly divided into two groups (n = 5 per group): a control group and a treatment group. Mice in the control group received intraperitoneal injections of 100 µL PBS, while those in the treatment group were administered 17AAG (20 µg/mouse, MCE, #HY-10211) intraperitoneally every two days for a total of 14 days. Tumor size was measured every two days using calipers, and volume was calculated using the formula: V = (length × width^2^)/2, where length and width represent the longest and shortest tumor diameters, respectively. At the experimental endpoint, tumors were harvested, weighed, and processed for immunohistochemical analysis.

### Immunohistochemistry

Paraffin-embedded tissue blocks were sectioned into 2.5-µm slices and transferred onto glass slides. The sections were immersed in 3% hydrogen peroxide to block endogenous peroxidase activity and incubated overnight at 4 ºC with primary antibodies. The sections were subsequently incubated with horseradish peroxidase-conjugated secondary antibodies (DakoCytomation, Glostrup, Denmark) at room temperature for 1 hour. The target gene expression was visualized via DAB staining, followed by hematoxylin counterstaining.

The tissue microarray (TMA) chips were obtained from Shanghai Weiaobio Biotechnology Co., Ltd (#ZL-PRC1801). Tissues were obtained from 90 patients. Formalin-fixed, paraffin-embedded (FFPE) tissue cores were punched and arrayed into recipient paraffin blocks. A tissue-array instrument (Beecher Instruments, Silver Spring, MD, USA) was used. Ninety paired sections of PCa tissues and matched adjacent tissues were sectioned and mounted on slides. Detailed clinical and pathological information of the TMA samples is provided in the Supplementary File (TMA).

### Immunofluorescence

Cells grown on glass coverslips were fixed with 4% paraformaldehyde at room temperature for 10 minutes. The cells were then washed twice with PBS. Blocking buffer (DakoCytomation, Glostrup, Denmark) was added for 30 minutes, followed by staining with primary antibodies and fluorescent secondary antibodies. The following primary antibodies were used: NDR1 (Santa Cruz Biotechnology, #sc-365555; ABclonal, #A8191), anti-AR (Cell Signaling Technology, #5153), and anti-USP9X (Proteintech, 55054-1-AP). The secondary antibodies used were anti-mouse IgG (Alexa Fluor 594 Conjugate) (Cell Signaling Technology, #8890) and anti-rabbit IgG (Alexa Fluor 488 Conjugate) (Cell Signaling Technology, #4412).

### CCK-8 cell proliferation assay

Cells in the logarithmic growth phase were selected and seeded into 96-well plates at a density of 3×10³ cells per well, with 100 µL of cell suspension per well. Each group included 3-5 replicates. After the cells had adhered to the plate, the culture medium was replaced, and 10 µL of 5 mg/mL CCK8 solution was added to each well. The cells were then incubated in the dark. After 2 hours, the medium was carefully removed from each well, and the absorbance was measured at 450 nm via a microplate reader.

### EdU proliferation assay

Cells in the logarithmic growth phase were selected and seeded into 6-well plates at a density of 1×10⁵ cells per well. After 24 hours of incubation, the cell adherence and conditions were observed. Before the EDU working mixture was added, 1 mL of cell culture medium was retained in each well. The EDU assay kit used for the experiment was purchased from Beyotime (#C0078S).

### Colony formation assay

Cells in the logarithmic growth phase were selected and seeded into 6-well plates at a density of 1,000 cells per well. The plates were placed in a cell incubator and cultured for approximately 14-28 days until cell colonies formed. Once colonies were established, the culture medium was removed, and the cells were washed with PBS. The cells were then fixed with 4% paraformaldehyde for 20 minutes. After the paraformaldehyde was removed, the cells were stained with 0.2% crystal violet for 5 minutes, followed by another PBS wash. The plates were air-dried and photographed, and the colonies were counted. The colony formation rate was calculated as follows: colony formation rate = (number of colonies/number of seeded cells) × 100%.

### Bioinformatics analysis

NDR1-related expression data were generated via the online platforms PIXdb (https://pixdb.org.uk/PIXdb/pages/index.php) and GEO2R (https://www.ncbi.nlm.nih.gov/geo/geo2r/). The predicted relationships between NDR1, AR, and USP9X were obtained from GEPIA 2.0 (http://gepia2.cancer-pku.cn/) and TIMER 2.0 (http://timer.comp-genomics.org/).

### Statistical analysis

Statistical analyses were performed via GraphPad Prism 9.0 software. The quantitative data from all the experiments are presented as the means ± standard deviations (SDs). One-way ANOVA or independent sample t tests were used to analyze differences between sample groups. A P value of < 0.05 was considered statistically significant. Adobe Illustrator CC, Adobe Photoshop CC, and ImageJ software were used for image processing and figure preparation.

## Supplementary Material

Supplementary figures and tables.

## Abbreviations

CRPC: Castration-resistant prostate cancer
PCa: prostate cancer
NDR1: nuclear Dbf2- related
AR: androgen receptor
ADT: androgen deprivation therapy
AR-Vs: AR splice variants
mCRPC: metastatic CRPC
PIXdb: prostate Integrative Expression Database
ENZR: enzalutamide resistance
CHX: cycloheximide
H&E: hematoxylin and eosin
ARSI: androgen receptor signaling inhibitor
ATCC: American Type Culture Collection
IHC: immunohistochemistry
