# Supplementary material for: NDR1 enhances USP9X-mediated AR deubiquitination and promotes enzalutamide resistance in castration-resistant prostate cancer
Source: Int J Biol Sci. 2025 Sep 3;21(13):5628–44. doi: 10.7150/ijbs.114686 (PMC12509700; doi:10.7150/ijbs.114686)
Supplement: Supplementary file 1 — Supplementary figures and tables. [file ijbsv21p5628s1.zip › Final-114686t1-Supplementary/Final-114686t1-Supplementary material.pdf]

NDR1 enhances USP9X-mediated AR deubiquitination and promotes enzalutamide resistance in castration-resistant prostate cancer

--Supplementary material

Zeyuan Zheng [1,†], Jinxin Li[2,†], Yifan Du [1,†], Liyan Li [1], Qingqing Wu [1], Bin Liu [1], Haodong Wu[1], Zeyi Zhang, Zuodong Xuan [1], Yue Zhao[1], Huimin Sun[3,*] and Chen Shao [1,*]

[1] Department of Urology, Xiang'an Hospital of Xiamen University, School of Medicine, Xiamen University, Xiamen 361101, China

[2] Department of Urology, Changhai Hospital, Naval Medical University (Second Military Medical University), Shanghai, China

[3] Central Laboratory, Xiang'an Hospital of Xiamen University, School of Medicine, Xiamen University, Xiamen 361101, China

[†] These authors contribute equally to this work.

[*]Correspondence:

Prof. Chen Shao (E-mail: cshao@xah.xmu.edu.cn)

Prof. Huimin Sun (E-mail: hmsun@xah.xmu.edu.cn)

1. Supplementary Figure

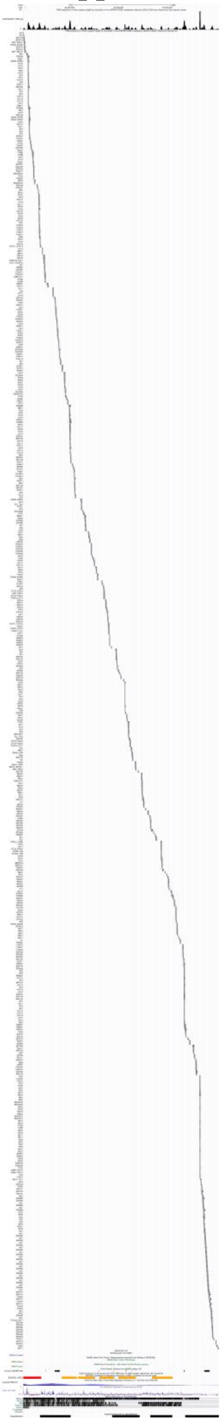

Figure S1. Bioinformatic prediction of AR binding sites in the upstream promoter region of NDR1. A 2000 bp region upstream of the transcription start site (TSS) of NDR1 was analyzed using the JASPAR 2024 core database via the UCSC Genome Browser (https://genome.ucsc.edu/). No canonical AR (androgen receptor) transcription factor binding sites were detected within this region. This supports the notion that NDR1 is not a direct transcriptional target of AR.

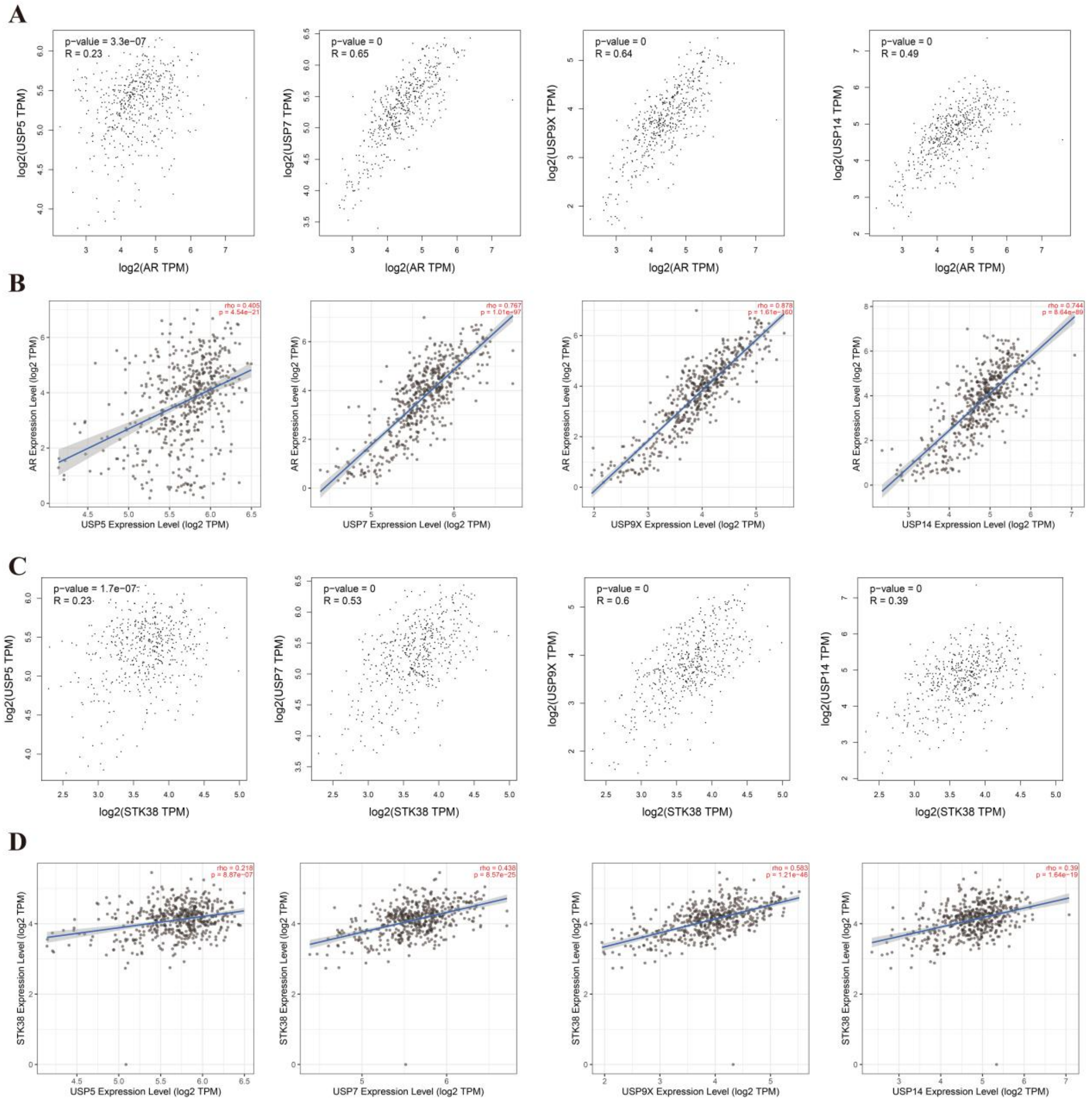

Figure S2. Correlation analysis between AR/NDR1 and DUBs (USP5, USP7, USP9X, USP14).

A: Correlation between AR and four DUBs (USP5, USP7, USP9X, USP14) in TCGA－PRAD dataset (GEPIA2). B: Correlation between DUBs and AR in TIMER2.0. C: Correlation between NDR1 and four DUBs in TCGA－PRAD dataset (GEPIA2). D: Correlation between DUBs and NDR1 in TIMER2.0.

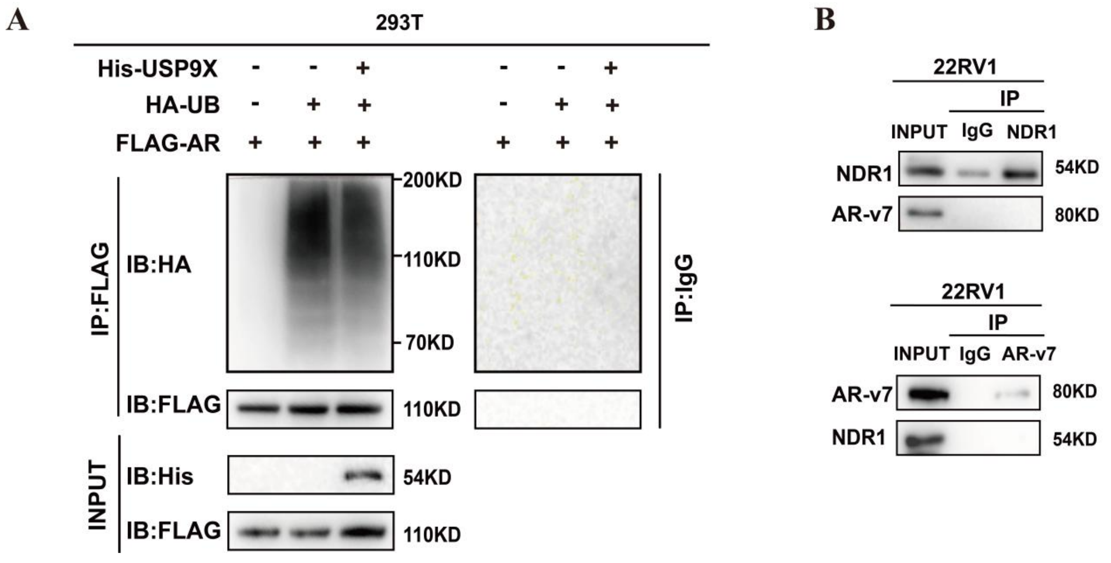

Figure S3. USP9X reduces AR ubiquitination, while NDR1 does not interact with AR-V7.

A: Ubiquitination assay in 293T cells co-transfected with FLAG-AR, HA-tagged ubiquitin (HA-UB), and His-tagged USP9X. AR ubiquitination was assessed by immunoprecipitation (IP) with anti-FLAG and immunoblotting (IB) with anti-HA. The presence of USP9X markedly decreased ubiquitinated AR, indicating that USP9X deubiquitinates AR. B: Co-immunoprecipitation assay in 22RV1 cells showed no detectable interaction between endogenous NDR1 and AR-V7, suggesting that NDR1 may not directly bind to AR-V7 in prostate cancer cells.

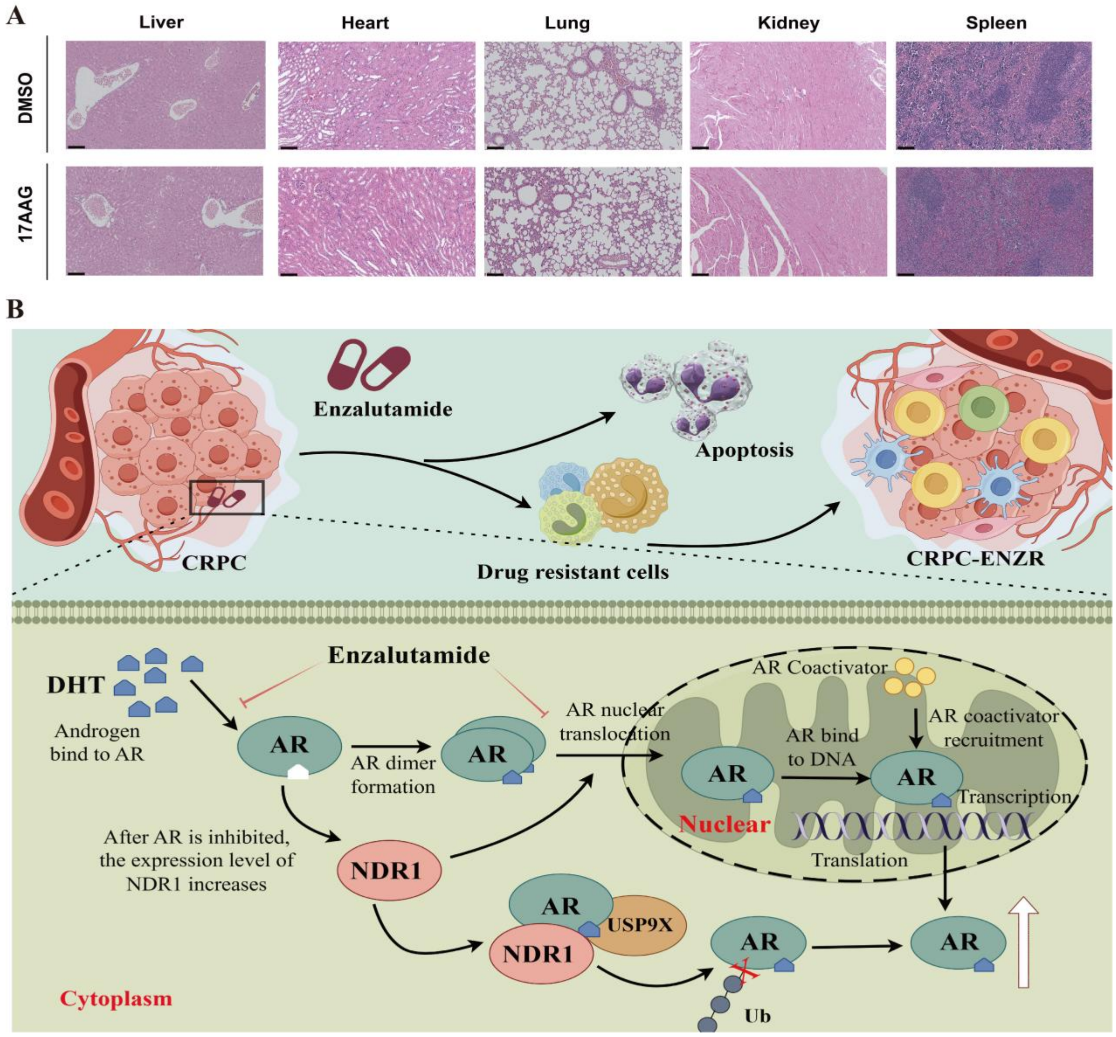

Figure S4. Histological safety profile and overall experimental mechanism model.

A: Representative H&E staining of major organs (liver, heart, lung, kidney, and spleen) from mice treated with DMSO or 17AAG, showing no significant histopathological alterations, suggesting low systemic toxicity of 17AAG (scale bar = 100 μ m). B: This figure depicts the mechanism by which NDR1 contributes to enzalutamide resistance in CRPC cells. In the early stages of enzalutamide treatment, AR inhibition leads to increased NDR1 expression. Elevated NDR1 levels subsequently increase the expression of USP9X, which deubiquitinates AR, thereby increasing AR protein stability. This stabilization of AR allows CRPC cells to evade the inhibitory effects of enzalutamide, ultimately promoting resistance.

2.Supplementary Table 1：Primers used in this study.

Gene	Sequence
Human AKR1C3 Forward	5’ GAGAAGTAAAGCTTTGGAGGTCACA 3’
Human AKR1C3 Reverse	5’ CAACCTGCTCCTCATTATTGTATAA 3’
Human AR-V7 forward	5’ CAGCCTATTGCGAGAGAGCT 3’
Human AR-V7 reverse	5’ GAAAGGATCTTGGGCACTTGC 3’
Human NDR1 forward	5’ GGTCGACATGGCAATGACAGGCTCAACA 3’
Human NDR1 reverse	5’ GCGCGTCTAGATTTTGCTGCTTTCATGTA 3’
Human AR Forward	5’ GGTTACACCAAAGGGCTAGAA 3’
Human AR Reverse	5’ GACTTGTAGAGAGACAGGGTAGA 3’
Human β-actin Forward	5’ CACCATTGGCAATGAGCGGTTC 3’
Human β-actin Reverse	5’ AGGTCTTTGCGGATGTCCACGT 3’

3. Supplementary Table 2：siRNAs used in this study.

	sense（5'-3'）	antisense（5'-3'）
Si-NC	UUCUCCGAACGUGUCACGUTT	ACGUGACACGUUCGGAGAATT
Si-STK38#1	GGGUGACAAUGACCAAAGUTT	ACUUUGGUCAUUGUCACCCTT
Si-STK38#2	CUGGAGUUGAGGAAAUAAATT	UUUAUUUCCUCAACUCCAGTT
Si-STK38#3	GGACAUGUGUAUGCAAUGATT	UCAUUGCAUACACAUGUCCTT
Si-AR#1	GCAGAAAUGAUUGCACUAUTT	AUAGUGCAAUCAUUUCUGCTT
Si-AR#2	CUGCUACUCUUCAGCAUUATT	UAAUGCUGAAGAGUAGCAGTT
Si-AR#3	CAGUCCCACUUGUGUCAAATT	UUUGACACAAGUGGGACUGTT
Si-USP7	ACCCUUGGACAAUAUUCCU	AGUCGUUCAGUCGUCGUAU
Si-USP9X	AAAUCGCUGGUAUAAAUUUTT	AAAUUUAUACCAGCGAUUUCU
