# Supplementary figures and images for: NDR1 enhances USP9X-mediated AR deubiquitination and promotes enzalutamide resistance in castration-resistant prostate cancer
Source: Int J Biol Sci. 2025 Sep 3;21(13):5628–44. doi: 10.7150/ijbs.114686 (PMC12509700; doi:10.7150/ijbs.114686)

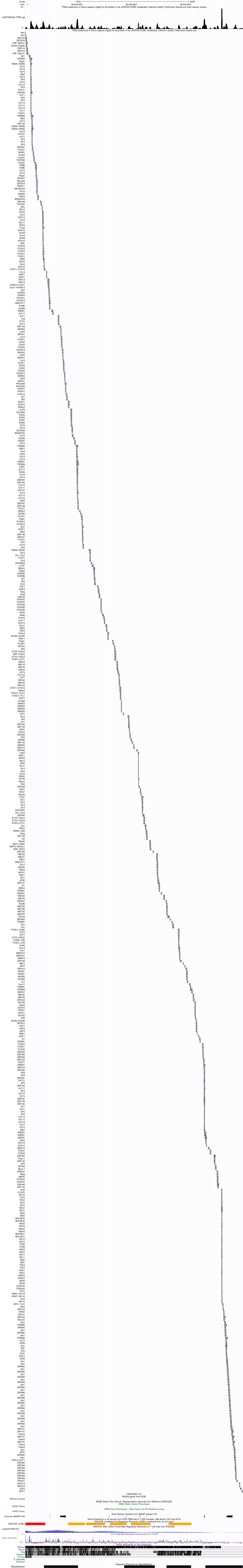

Supplement: Supplementary file 1 — Supplementary figures and tables. [file ijbsv21p5628s1.zip › Final-114686t1-Supplementary/Fig.S1.png]

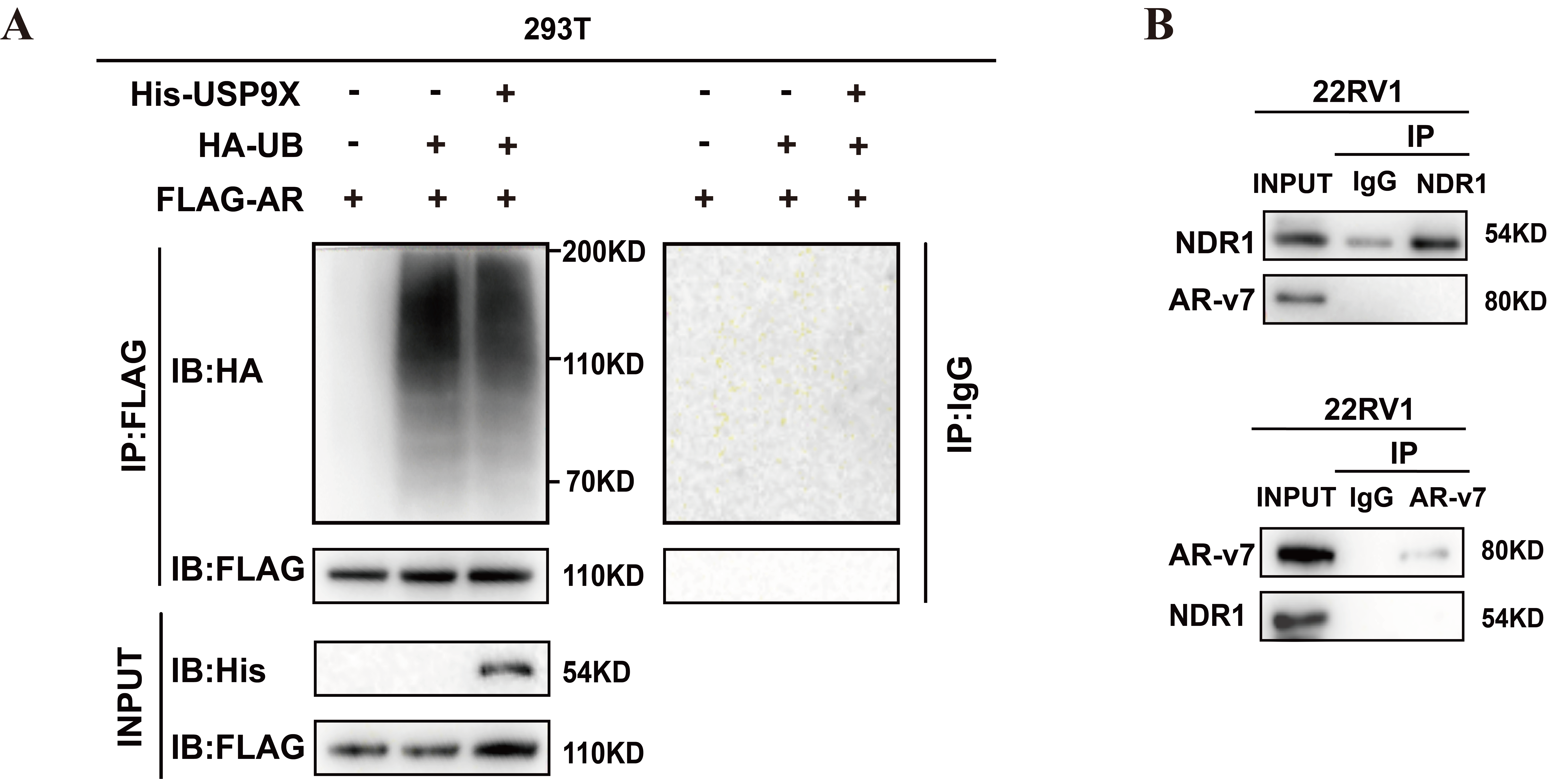

Supplement: Supplementary file 1 — Supplementary figures and tables. [file ijbsv21p5628s1.zip › Final-114686t1-Supplementary/fig.S3.png]
